# Developmental divergence: motor trajectories in children with fragile X syndrome with and without co-occurring autism

**DOI:** 10.1186/s11689-019-9281-1

**Published:** 2019-10-05

**Authors:** Elizabeth A. Will, Somer L. Bishop, Jane E. Roberts

**Affiliations:** 1 Department of Psychology, University of South Carolina, 1512 Pendleton Street, Columbia, SC 29208 USA; 2 Department of Psychiatry, University of California San Francisco, 401 Parnassus Ave., San Francisco, CA 94143 USA

**Keywords:** Developmental trajectories, Fragile X syndrome, Autism spectrum disorder, Motor development

## Abstract

**Background:**

Autism spectrum disorder (ASD) is highly prevalent in fragile X syndrome (FXS), affecting 50–70% of males. Motor impairments are a shared feature across autism and FXS that may help to better characterize autism in FXS. As motor skills provide a critical foundation for various language, cognitive, and social outcomes, they may serve an important mechanistic role for autism in FXS. As such, this study aimed to identify differences in motor trajectories across direct assessment and parent-report measures of fine and gross motor development between FXS with and without autism, and typical development, while controlling for cognitive functioning.

**Methods:**

This prospective longitudinal study included 42 children with FXS, 24 of whom also had ASD (FXS + ASD), as well as 40 typically developing children. The Mullen Scales of Early Learning provided a direct measure of fine and gross motor skills, and the Vineland Adaptive Behavior Scales provided a measure of parent-reported fine and gross motor skills. Random slopes and random intercepts multilevel models were tested to determine divergence in developmental motor trajectories between groups when controlling for cognitive level.

**Results:**

Model results indicated the children with FXS + ASD diverged from TD children by 9-months on all measures of gross and fine motor skills, even when controlling for cognitive level. Results also indicated an early divergence in motor trajectories of fine and gross motor skills between the FXS + ASD and FXS groups when controlling for cognitive level. This divergence was statistically significant by 18 months, with the FXS + ASD showing decelerated growth in motor skills across direct observation and parent-report measures.

**Conclusions:**

This study is the first to examine longitudinal trends in motor development in children with FXS with and without comorbid ASD using both direct assessment and parent-report measures of fine and gross motor. Furthermore, it is among the first to account for nonverbal cognitive delays, a step towards elucidating the isolated role of motor impairments in FXS with and without ASD. Findings underscore the role of motor impairments as a possible signal representing greater underlying genetic liability, or as a potential catalyst or consequence, of co-occurring autism in FXS.

## Background

Autism spectrum disorder (ASD) affects 50–70% of males with fragile X syndrome [1–4] (FXS), which is the leading heritable cause of intellectual disability (ID) [5]. FXS is an X-linked disorder caused by a mutation in the *Fragile X Mental Retardation 1* (FMR1) gene [6, 7] that affects approximately 1 in 4000 to 8000 individuals [8, 9]. There are many shared features between FXS and ASD, including impairments in language, social communication, and adaptive functioning; however, there is an important phenotypic distinction as well. For example, virtually all individuals with FXS experience ID [9], compared to only approximately 30% of individuals with ASD [10]. Several decades of research highlight the complex relationships between ASD and FXS, with some debate as to whether ASD should be conceptualized as part of the FXS phenotype, versus distinct comorbidity [11]. Most of this debate has focused on children and adolescents with FXS and/or ASD; however, prior work on ASD in young children with FXS [12, 13] suggests that a subset of children with FXS do in fact follow a differentiable developmental trajectory consistent with a clinical diagnosis of ASD. Nevertheless, many questions remain about the nature of ASD in FXS, particularly during early development. As such, prospective longitudinal examination of infants with FXS provides opportunities for increased understanding of the onset and early developmental course of ASD among children with increased genetic liability for ASD.

Motor impairments are commonly reported among individuals with non-syndromic ASD (nsASD, ASD unassociated with FXS or any other particular syndrome) [14] as well as in FXS, irrespective of comorbid outcomes [15]. In typical development, motor skills provide a critical developmental foundation for many of the abilities that are impaired in ASD, including language and communication [16], imitation, attentional control [17], and social cognition [18–21]. Early motor experiences afford greater opportunities for interaction with the environment, the development of representations of the self and others as agents, and an expansion of one’s attention and communication repertoire [20–22]. Accordingly, emergent evidence suggests that early motor impairments in some children may contribute to the development or enhanced severity of certain ASD features [19, 21, 23–25].

Gross motor skills are in fact associated with social communication and language development in ASD and high-risk populations [23, 26, 27]. For example, high-risk infants who have acquired independent walking have better social communication skills than high-risk infants of the same age and cognitive level who are not yet walking [23]. For these infants, motor delays may directly influence social communication skills; or, alternatively, these infants may experience a propensity for both motor and social communication delays resulting from added genetic liability [28]. Recent work substantiates a link between motor impairments and increased genetic liability in some children with ASD [29, 30]. Specifically, delayed onset of independent walking in children with ASD ascertained via the Simons Simplex Collection (SSC) is associated with an increased likelihood of an ASD-associated de novo mutation [29, 30]. Further delineation of motor impairments in FXS may therefore contribute to an enhanced understanding of the link between genetic liability, motor impairment, and ASD.

Motor impairments emerge during infancy in FXS and, unlike most cases of ASD, are often one of the first notable signs of atypical development, with parents reporting increased atypical motor behaviors [31] and delayed motor skill acquisition [15, 32]. Fine motor skills diverge from typical development as early as 6 [33] and 9 months in FXS [34], and impairments in fine, as well as gross motor, persist across development [13, 33, 35, 36]. Motor impairments may be even more pronounced in children with co-occurring FXS and ASD [12, 32, 35], as parent-report gross motor skills are significantly more delayed in children with FXS with ASD than those with FXS only [32]. In addition, motor impairments have emerged as the most salient predictor in the infancy of a later diagnosis of ASD in FXS [12]. On direct assessment measures, fine motor skills are associated with ASD severity in children with FXS between 8 and 68 months old [34], and motor impairments observed via direct assessment in 12-month-old infants predict ASD outcomes at 24 months of age in FXS [12]. Thus, it is possible that motor impairments may also serve as an early sign of co-occurring ASD in FXS.

A major challenge in this area of research is that motor and cognitive development are inextricably linked, informing one another across development [20, 37–40]. In addition, motor and cognitive ability are more closely related in populations with ID relative to typical development [40]. Thus, the role of motor vulnerabilities as a unique facilitator or indicator of the development of ASD in FXS, independent of overall cognitive impairments characteristic of FXS, has not been firmly established. For example, Zingerevich et al. [35] (2009) found that differences in directly observed fine motor skills in children with FXS between 12 and 76 months old with and without co-occurring ASD were accounted for by differences in visual reception skills. Furthermore, direct assessment of fine and gross motor skills in younger children (i.e., 21 and 48 months) with FXS indicated no significant differences as a function of ASD comorbidity, but both FXS groups showed significantly poorer motor repertoires than children with ASD without a genetic diagnosis [13].

With growing evidence that early trajectories may provide insights into different etiologies of ASD, it is critical to understand the role of motor impairments in ASD in FXS while also considering cognitive abilities. Thus, the current study aims to identify potential differences in developmental trajectories in *both* gross and fine motor skills between infants and young children with FXS with and without ASD. In addition, we examine motor impairments across *both* direct assessment and parent-report measures to capture the unique information about motor development provided by these two sources. Furthermore, we aim to isolate motor impairments as a potential mechanism of ASD by controlling for nonverbal cognitive abilities using a nonverbal developmental quotient (NVDQ).

Our specific research questions are as follows:

1. At what point do fine and gross motor trajectories in children with FXS, with and without ASD, diverge from typical development?
2. Do early trajectories of fine and gross motor development differ between children with FXS who do and do not go onto be diagnosed with ASD?

## Methods

### Participants

This prospective longitudinal study included 42 children with FXS (mean chronological age (CA) = 15.66 months at initial enrollment), and 40 TD children (*M* CA = 9.54 months at initial enrollment) between 6 and 60 months old. The FXS group included 24 children with FXS + ASD (*M* CA = 19.13 at initial enrollment) and 18 children with FXS only (*M* CA =12.20 at initial enrollment). Table 1 presents full participant characteristics. In total, there were 282 observations across the sample, with 70 in the FXS only group, 71 in the FXS + ASD group, and 141 in the TD group. Inclusion in the FXS group was determined by positive FXS diagnosis (i.e., > 200 repeats of the CGG sequence on the FMR1 gene) per diagnostic report. TD participants were included based on the absence of a developmental delay and autism diagnosis, verified by our assessment protocol and clinical team, as well as lack of a first-degree relative with an ASD diagnosis. The groups were well matched on average chronological age across assessments (*p* = .59). Participants were part of a larger prospective longitudinal study on early development in FXS at the University of South Carolina.

**Table 1:** Participant Demographics

	FXS-Whole Group (n=42)	FXS+ASD (n=24)							FXS-only (n=18)							TD (n=40)						
Assessment Age (months)	-	9	12	18	24	36	48	60	9	12	18	24	36	48	60	9	12	18	24	36	48	60
Participant n	-	11	12	2	20	8	9	3	12	15	1	18	11	7	2	37	38	0	32	15	15	4
	^*^M (SD)	^*^M (SD)							^*^M (SD)							^*^M (SD)						
Chronological Age (months)	24.31 (14.74)	24.77 (15.19)							23.86 (14.38)							22.20 (14.56)						
Nonverbal Dev. Quotient	72.39 (24.84)	61.76 (20.73)							83.02 (24.17)							104.73 (17.99)						
Direct Assessment																						
Gross Motor	16.58 (6.42)	15.78 (5.74)							17.30 (6.96)							18.67 (6.06)						
Fine Motor	17.59 (7.87)	15.67 (6.54)							19.51 (8.63)							22.43 (10.60)						
Parent Report																						
Gross Motor	33.91 (19.89)	30.89 (18.89)							37.21 (20.76)							40.35 (20.88)						
Fine Motor	16.63 (8.91)	14.56 (7.31)							18.89 (9.97)							21.70 (11.25)						
	N (%)	N (%)							N (%)							N (%)						
Male	30 (71.4)	22 (91.67)							8 (44.44)							32 (80.00)						
Caucasian	26 (61.91)	17 (70.83)							9 (50.00)							33 (82.50)						
Black	3 (7.14)	1 (4.16)							2 (11.11)							4 (10.00)						
Other/Multiracial	13 (30.95)	6 (25.00)							7 (38.89)							3 (7.50)						

^*^Sample grand M (SD) across assessments

### Measures

#### Gross and fine motor skills—direct assessment

The Mullen Scales of Early Learning [41] is a standardized measure of early childhood development normed for ages 0–68 months. The MSEL measures development across the following domains: expressive language, receptive language, visual reception, fine motor, and gross motor, each of which has mean standard scores of 50 and standard deviation of 10. An early learning composite can also be derived and has a mean of 100 and a standard deviation of 15. Raw scores from the fine and gross motor domains were used in all analyses due to the floor effects of standard scores in ID populations. The gross motor domain measures skills such as rolling over and independent sitting in infancy, as well as navigating stairs and running smoothly at preschool ages. The fine motor domain measures skills including reaching and grasping in infancy, as well as copying shapes (visuomotor integration) and stacking blocks at preschool ages. Because fine motor scores factor into the Early Learning Composite (ELC) and nonverbal mental age derivative, which is typically the averaged fine motor and visual reception age equivalent scores, we derived a developmental quotient from the visual reception (VR) domain to control for cognitive functioning while avoiding statistical collinearity issues. The developmental quotient was computed using VR age equivalent score and the following equation: $\frac{\mathrm{VR}\ \mathrm{age}\ \mathrm{equivalent}}{\mathrm{chronological}\ \mathrm{age}}\times 100$. Visual reception developmental quotients were used in all statistical models to control for the effect of cognitive development on motor skill acquisition.

#### Gross and fine motor skills—parent report

The Vineland Adaptive Behavior Scales—2nd Edition [42] is a semi-structured caregiver interview that measures adaptive functioning level across four domains: communication, socialization, daily living skills, and motor skills. Items are scored on a 0–2 scale indicating the consistency with which the individual independently completes the assessed skill: (0) never, (1) sometimes, or (2) usually. All standard scores have a mean of 100 and a standard deviation of 15 and each domain standard score comprises the Adaptive Behavior Composite (ABC). The gross motor domain includes items such as pulling to sitting, and independent standing and walking during infancy and toddlerhood, as well as jumping and hopping at preschool ages. The fine motor domain measures items such as transferring items between hands during infancy, as well as turning pages and doorknobs at preschool ages. Raw scores from the fine and gross motor subdomains were used in all analyses.

#### ASD diagnostic status

Information from the Autism Diagnostic Observation Schedule-2nd Edition (ADOS-2) [43], a semi-structured direct observation of ASD symptoms, and the Autism Diagnostic Interview–Revised [44], a semi-structured parent interview of ASD symptoms, were used in conjunction with all other available information to determine whether a clinical best estimate (CBE) diagnosis of ASD was appropriate (see below).

### Procedures

Study approval was obtained at the University of South Carolina. Participants were recruited through local and national (FXS) organizations. Participants were assessed between 6 and 60 months of age. Assessment ages varied, but the majority were assessed at regular intervals of 9, 12, 24, 36, 48, and 60 months. Table 1 presents the number of participants assessed at each major age interval.

As part of a larger assessment battery for the longitudinal study, all participants (6-months-old and older) completed the Mullen Scales of Early Learning [41], as well as the ADOS-2 [43] beginning at 24 months. Gross motor direct assessment was added later to the study protocol, resulting in some Mullen Gross Motor missing at random. This was accounted for in the analytic approach. Participants’ parents completed interviews for the Vineland Adaptive Behavior Scales–Survey Interview Form–Second Edition [42]. Clinical best estimate (CBE) procedures were implemented to determine ASD status across all groups at 36-months or older. CBE diagnosis was made from a thorough review of scores from the parent and child assessments, review of ADOS tapes, and expert clinical judgment by a licensed psychologist and certified ADOS trainer, as well as trained research staff reliable in ADOS administration. For the current study, if ADOS data were not available at a 36-month or older assessment, 24-month assessment CBE diagnoses were used (*n* = 2). Prior work indicates diagnostic stability in children receiving an ASD diagnosis prior to 36 months [45]. As such, the two participants with only a 24-month diagnostic outcome were retained in the FXS + ASD group. Of the overall FXS sample, 24 of 42 participants (57%) met criteria for comorbid ASD (FXS + ASD).

### Analytic approach

Multilevel modeling was used to estimate gross and fine motor trajectories across groups and test age-by group interactions to determine a point of divergence between groups controlling for cognitive level (i.e., VRDQ). The point in divergence was determined to be the age at which groups significantly differed in their motor skills. Because initial individual differences and individual variation was expected, random intercepts and random slopes models were used, which also appropriately account for missing data. Due to floor effects with standard scores, raw scores were used in all analyses. In the initial models, age was centered at 9-months, the approximate age of initial study enrollment of most participants for primary model testing. We probed age-by-group interactions within a multilevel framework by re-centering age at subsequent ages of assessment (e.g., 12 and 18 months) to yield predicted points of developmental divergence (i.e., statistically significant difference) between groups [46]. We probed interactions at an 18-month interval to yield predicted estimates of motor skills at 18-months as a prototypical value [47]. This approach provides estimates of modeled group comparisons at various ages, with age as a continuous predictor, while accounting for the repeated assessments across participants.

## Results

### Gross motor

**Fig. 1:**
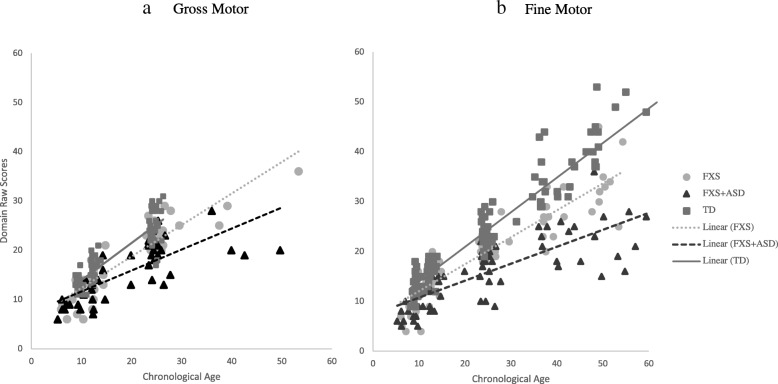
Direct assessment motor trajectories

Results showed that after controlling for NVDQ, children with FXS + ASD significantly diverged in their gross motor trajectories from TD controls by 9-months (*b* = 2.27; *p* < .001), and this divergence became greater over time (see Fig. 1a). These findings were also consistent across parent-report measures of gross motor, with FXS + ASD significantly diverging from TD controls in their motor trajectories by 9-months (*b* = 6.52; *p* = .003), and this divergence became greater over time (see Fig. 1a). Controlling for NVDQ, those with FXS without ASD also significantly diverged from TD controls at 9 months in direct assessment (*b* = 1.57; *p* = .039). However, differences in parent-report gross motor were trending towards significance at 9 months (*b* = 3.60; *p* = .08) and reached significance by 18 months (*b* = 3.86; *p* = .027).

**Fig. 2:**
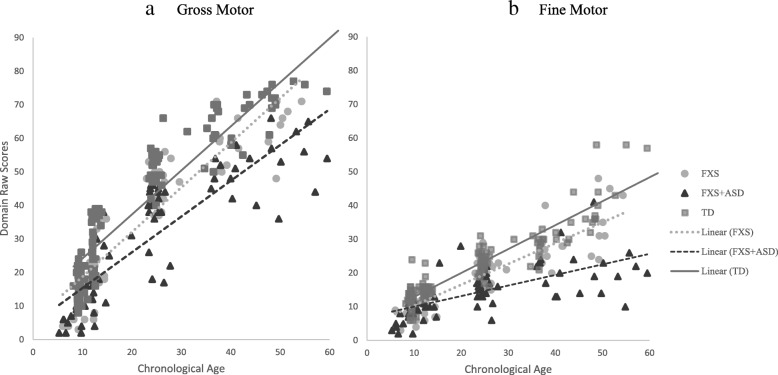
Parent-report motor trajectories

Controlling for NVDQ, the FXS + ASD and FXS-only groups significantly diverged in their gross motor trajectories at 18-months (*b* = 1.90; *p* = .02), at which point this divergence became greater over time. This pattern of divergence was also consistent across trajectories of parent-report gross motor development, with statistically significant differences identified at 18-months (*b* = 4.49; *p* = .022) and becoming more divergent over time (Fig. 2a).

### Fine motor

The FXS + ASD group showed significant divergence in their direct assessment trajectories of fine motor from TD controls by 9 months (*b* = 1.30; *p* = .049), which became greater over time (see Fig. 1b). This trend was also evident in trajectories of parent-report fine motor skills, with significant divergence occurring by 9-months (*b* = 2.65; *p* = .004) and growing over time. Controlling for NVDQ, trajectories in direct assessment fine motor also began to diverge between the FXS-only and TD controls by 12 months (*b =* 1.03; *p* = .065) and was statistically significant by 18 months (*b* = 1.95; *p* < .001). Interestingly, parent-report fine motor skills in FXS only were significantly different from the TD group by 9 months (*b* = 2.01; *p* = .02) and these differences became greater over time (see Fig. 2b).

Controlling for NVDQ, the FXS + ASD and FXS-only groups demonstrated statistically significant differences in fine motor skills by 18 months (*b* = 1.68; *p* = .006). At this point in development, the FXS-only group scored approximately 2-points higher on fine motor relative to those with FXS + ASD, and this divergence became greater over time. These trends were also consistent across trajectories of parent-report fine motor development when controlling for NVDQ. Specifically, by 18 months, even controlling for NVDQ, the FXS group was predicted to perform 2-points higher than the FXS + ASD group (*b* = 2.15; *p* = .021) and divergences became greater over time.

### Summary of findings

Results indicate that fine and gross motor skill acquisition is impaired in children with FXS + ASD relative to FXS -only and TD children at early ages. Differences emerged between the FXS + ASD and FXS-only groups by 18 months. As expected, there was an early divergence from typical development in both the FXS + ASD and FXS-only group, though this contrast emerged earlier for the FXS + ASD group on both direct and parent-report measures. There was remarkable consistency in patterns of divergence in motor trajectories across direct assessment and parent-report measures for contrasts between the FXS + ASD and FXS-only groups, and the FXS + ASD and typical groups, but less consistency for contrasts between the FXS-only and TD group. Full model results are presented in Table 2 (gross motor) and Table 3 (fine motor).

**Table 2:** Gross motor trajectory models

	Gross motor direct assessment								
	9-month models			12-month models			18-month models		
	*b*	SE(*b*)	*p*	*b*	*S*E(*b*)	*p*	*b*	SE(*b*)	*p*
Intercept	8.63	0.98	< .001	10.43	0.90	< .001	14.03	0.81	< .001
VRDQ	0.02	0.01	.017	0.02	0.01	.017	0.02	0.01	.017
Age	0.60	0.06	< .001	0.60	0.06	< .001	0.60	0.06	< .001
FXS	0.71	0.91	.441	1.11	0.80	.169	1.91	0.77	.016
TD	2.21	0.85	.013	2.71	0.76	.001	3.72	0.75	< .001
FXS × age	0.13	0.08	.110	0.13	0.08	.110	0.13	0.08	.110
TD × age	0.17	0.08	.029	0.17	0.08	.029	0.17	0.08	.029
	Gross motor parent report								
	9-month models			12-month models			18-month models		
	*b*	SE(*b*)	*p*	*b*	SE(*b*)	*p*	*b*	SE(*b*)	*p*
Intercept	10.97	2.55	< .001	14.39	2.42	< .001	21.24	2.22	< .001
VRDQ	0.05	0.03	.077	0.05	0.03	.077	0.05	0.03	.077
Age	1.14	0.03	< .001	1.14	0.08	< .001	1.14	0.08	< .001
FXS	2.92	2.35	.218	3.45	2.17	.117	4.49	1.92	.022
TD	6.52	2.14	.003	7.13	2.02	.001	8.34	1.89	< .001
FXS × age	0.17	0.11	.122	0.17	0.11	.122	0.17	0.11	.122
TD × age	0.20	0.10	.039	0.20	0.10	.039	0.20	0.10	.039

**Table 3:** Fine motor trajectory models

	Fine motor direct assessment								
	9-month models			12-month models			18-month models		
	*b*	SE(*b*)	*p*	*b*	SE(*b*)	*p*	*b*	SE(*b*)	*p*
Intercept	5.88	0.78	< .001	7.22	0.74	< .001	9.90	0.69	< .001
VRDQ	0.06	0.01	< .001	0.06	0.01	< .001	0.06	0.01	< .001
Age	0.45	0.03	< .001	0.45	0.03	< .001	0.45	0.03	< .001
FXS	0.73	0.71	.305	1.05	0.64	.107	1.68	0.60	.006
TD	1.30	0.65	.049	2.08	0.61	.001	3.63	0.59	< .001
FXS × age	0.11	0.05	.033	0.11	0.05	.032	0.11	0.05	.032
TD × age	0.26	0.04	< .001	0.26	0.04	< .001	0.26	0.04	< .001
	Fine motor parent report								
	9-month models			12-month models			18-month models		
	*b*	SE(*b*)	*p*	*b*	SE(*b*)	*p*	*b*	SE(*b*)	*p*
Intercept	6.93	1.12	< .001	8.19	1.07	< .001	10.71	1.03	< .001
VRDQ	0.03	0.01	.024	0.03	0.01	.024	0.03	0.01	.024
Age	0.42	0.05	< .001	0.42	0.05	< .001	0.42	0.05	< .001
FXS	0.64	0.97	.511	1.14	0.91	.215	2.15	0.92	.022
TD	2.65	0.90	.004	3.39	0.86	< .001	4.86	0.89	< .001
FXS × age	0.17	0.07	.012	0.17	0.07	.012	0.17	0.07	.012
TD × age	0.25	0.06	< .001	0.25	0.06	< .001	0.25	0.06	< .001

## Discussion

The present study takes an initial step towards characterizing the role of motor impairments and their implications for ASD in FXS. This study is the first to examine divergence in motor trajectories between children with FXS with and without comorbid ASD across direct observation and parent-report measures of both fine and gross domains of motor development. Furthermore, this study is also among the first to account for delays in cognitive development, a construct closely related to motor development, using a measure of nonverbal cognition that is relatively independent of motor skills.

Trajectories of children with FXS with co-occurring ASD diverged from those of typically developing children in both gross and fine motor domains by 9 months old, and these differences were consistent across direct assessment and parent-report measures (see also ref. [12, 32]). Interestingly, the FXS-only group began to show differences by 9 months, but only directly assessed gross motor and parent-report fine motor differences were statistically significant prior to 12 months. We also found significant divergence in fine and gross motor trajectories between FXS + ASD and FXS early in life, even after accounting for general cognitive delays. Infants with FXS + ASD showed decelerated fine and gross motor development compared to those with FXS-only, resulting in lower skills as early as 12 months, differences which reached statistical significance by 18 months and became greater over time. Trajectories diverged even when controlling for NVDQ, indicating that motor differences could not be fully explained by greater cognitive delays in those with FXS and ASD. These results provide initial insight into fundamental differences in the motor skills of those with FXS + ASD and those with FXS-only, indicating a potential key role of motor in the development of ASD in children with FXS.

As this is a first step in identifying differences in motor repertoires of children with FXS who go on to develop ASD versus those that do not, unanswered questions regarding these motor differences remain. Motor may serve as a catalyst for a disrupted developmental cascade [48], leading to an enhanced presentation of ASD features for some children with FXS, who already have an underlying vulnerability to develop ASD. That is, early motor impairments may place constraints on other developmental processes, specifically attention, that lead to less optimal social [18, 23] and cognitive [37, 49–51] outcomes evident in those with FXS and ASD [12, 13]. Given that motor development is a self-refining process that requires system-level organization along with input from one’s environment [20], motor delays with concurrent ASD and additional genetic liability may enact a multiplicative effect [52], resulting in diminished developmental outcomes for those with FXS and ASD. The disruption in this developmental pathway may account for generally lower cognitive abilities in those with FXS and ASD compared to those with FXS-only [13]. The timing and persistence of motor differences as a function of ASD status across both direct assessment and parent-report measures suggests that motor impairments may also play a vital role in development for children with FXS and ASD, particularly related to language [26, 27], social communication [23], and adaptive outcomes [53]. It is also possible that poorer motor skills in the FXS + ASD partially resulted from, or were made even worse by having ASD. For example, deficits in imitation skills and reduced motivation to perform certain activities could have negatively affected the acquisition of more advanced motor skills for children with FXS and ASD.

There is an additional possibility that motor impairments identified in those with FXS with ASD signal rare genetic mutations not present in those with FXS only [29, 30]. Additional damaging de novo mutations are associated with motor impairments in children with ASD [29, 30], and these associations are the target of future research. Thus, it is possible that motor impairments in those with FXS and ASD reflect an undetected genetic etiology [30]. Characterizing potential underlying de novo mutations in the present sample was beyond the scope of this study and further work is necessary to determine underlying etiological mechanisms of motor impairments in FXS, in general, but particularly in those with FXS and ASD.

### Limitations

Although the present study was the first to examine prospective longitudinal trajectories of both gross and fine motor development in children with FXS with and without ASD across direct assessment and parent report, it is not without limitations. One limitation includes the lack of comparison to another neurogenetic group also at risk for ASD. Such comparisons can yield better insight into the role of motor impairments in the development of ASD by elucidating what impairments may exist as a feature of neurogenetic etiology versus ASD risk with greater specificity than in FXS alone. Another potential limitation is the focus on motor development specifically, rather than other aspects of motor impairments such as motor stereotypies, which should be included in future work on FXS and concurrent ASD as this may also further delineate these groups [31]. An additional limitation relates to the measurement of motor skills while accounting for general cognitive ability. Assessment of motor abilities is somewhat constrained by currently available measures, designed to assess broad development rather than precise motor skills. While additional work is necessary to more carefully characterize the nature of motor impairments in FXS, longitudinal examination of gains in motor abilities over time contribute to our fundamental understanding of motor impairments in FXS and their role in the presence of concurrent FXS and ASD. In addition, although a close estimate of cognitive functioning, the visual reception domain of the MSEL is not a comprehensive estimate of cognitive level and requires motor planning in some responses. Future work to develop more precise motor measures may circumvent collinearity issues with other measures of the cognitive level. Finally, although the present sample size is quite robust considering the prevalence of FXS, it may impose limits on the statistical power to detect small to medium effects. Furthermore, our sample was variable across age intervals, and relatively sparse at some ages. As such, findings related to the timing of developmental divergence in motor trajectories between FXS and FXS + ASD identified in the present study are, in some cases, based on relatively small samples. Future work should aim for replication in larger samples.

### Future directions and conclusions

Increased attention has focused on characterizing the nature and role of motor impairments in ASD. Examining prospective longitudinal trajectories of motor development in an etiologically distinct genetic subgroup of ASD, such as FXS, can further our understanding of what role motor impairments serve as a catalyst or consequence of ASD, or alternatively, a result of additional genetic risk, ID, and/or ASD. The present study findings suggest that divergence in motor development may occur independently of cognitive impairment and therefore contribute to and/or serve as a marker of concurrent ASD in FXS. Future work should aim to identify the direction of influence between motor and other important areas of development, such as cognition, language, and attention, as well as the underlying mechanisms and long-term consequences of motor impairments in FXS. These efforts may provide insight into the role of motor impairments as a catalyst or outcome of ASD-related risk in FXS.

## Data Availability

The datasets used and/or analyzed during the current study are available from the corresponding author on reasonable request.

## Abbreviations

ASD: Autism spectrum disorder
CA: Chronological age
FXS: Fragile X syndrome
M: Mean
MSEL: Mullen Scales of Early Learning
TD: Typically developing
VABS-II: Vineland Adaptive Behavior Scales–2nd Edition
